# Optimizing Regional Access to Extracorporeal Cardiopulmonary Resuscitation: A Geographic-Information-System-Based Comparison of Hospital- and Prehospital-Initiated Strategies in Nara Prefecture, Japan

**DOI:** 10.3390/healthcare14121762

**Published:** 2026-06-18

**Authors:** Arisa Kinoshita, Hideki Asai, Yasuyuki Kawai, Keita Miyazaki, Koji Yamamoto, Hirozumi Okuda, Hidetada Fukushima

**Affiliations:** Department of Emergency and Critical Care Medicine, Nara Medical University, Shijo-cho, 840, Kashihara City 634-8522, Nara, Japan; arisakinoshita99@naramed-u.ac.jp (A.K.);

**Keywords:** emergency medical services, prehospital care, physician-staffed ambulance, computer simulation

## Abstract

**Highlights:**

**What are the main findings?**

- This geographic-information-system-based simulation study demonstrated that a physician-staffed ambulance strategy for prehospital extracorporeal cardiopulmonary resuscitation (ECPR) could achieve broader geographic coverage compared with expanding hospital-based ECPR facilities, without requiring additional fixed hospital infrastructure.
- However, sensitivity analysis revealed that this geographic advantage was contingent on achieving on-scene procedures within the assumed time parameters.

**What are the implications of the main findings?**

- A mobility-focused prehospital ECPR model extended potential access beyond what was achievable through hospital expansion, particularly in resource-limited areas.
- Optimization of prehospital deployment may represent a geographically feasible approach to expanding ECPR access in mixed urban–rural regions**, though operational feasibility and cost-effectiveness require further evaluation before clinical implementation.

**Abstract:**

**Background**: Extracorporeal cardiopulmonary resuscitation (ECPR) can improve outcomes following refractory out-of-hospital cardiac arrest (OHCA); however, access is constrained by geography and resources. This study compared two strategies against the current system in Nara Prefecture, Japan: a two-stage hospital model using chest-pain network hospitals as ECPR-initiation sites, and a prehospital ECPR model using physician-staffed ambulances from two extracorporeal membrane oxygenation (ECMO)-ready hospitals. **Methods**: A geographic information system (GIS)-based simulation was conducted using emergency medical service (EMS) records of witnessed cardiac-origin OHCA cases (2017–2022). Isochrone analyses estimated areas reachable within a 60 min arrest-to-ECMO target. In the two-stage hospital model, patients located within a 15 min transport radius from chest-pain network hospitals were considered geographically covered. In the prehospital ECPR model, a physician-staffed ambulance was assumed to reach arrest sites within a 25 min travel-time radius from ECMO-ready hospitals. The study outcome was geographic coverage, defined as the proportion of cases within each service area; the two strategies were compared using McNemar’s test for paired proportions. **Results**: Among 1476 included cases, the coverage rate was as follows: current system, 28.7%; two-stage hospital model, 65.2%; prehospital model, 70.4% (*p* < 0.001). Certain eastern and southern mountainous regions remained outside both coverage areas. **Conclusions**: Using real-world EMS data, a mobility-focused prehospital ECPR strategy provided broader potential geographic access without requiring additional fixed hospital infrastructure than expanding hospital-based initiation sites. Optimization of prehospital deployment may represent a geographically feasible approach to expanding ECPR access in mixed urban–rural regions, though operational feasibility and cost-effectiveness require further evaluation.

## 1. Introduction

Out-of-hospital cardiac arrest (OHCA) is a major global public health issue with extremely high mortality rates. In Europe, approximately 350,000–700,000 cases occur annually, and in North America, 300,000–400,000 cases, with the majority resulting in death [1,2]. In Japan, approximately 130,000 OHCAs occur each year, with a one-month survival rate of only 8% and a favorable neurological outcome achieved in just 3.6% of cases [3,4]. Despite widespread availability of automated external defibrillators (AEDs), increased rates of bystander cardiopulmonary resuscitation (CPR), and advanced procedures by emergency medical services (EMS), outcomes of OHCA remain dismal.

Extracorporeal cardiopulmonary resuscitation (ECPR) has recently attracted attention as a potential lifesaving intervention for refractory OHCA, with evidence suggesting improvements in both survival [5,6] and neurological outcomes [7,8,9,10,11,12,13]. However, hospitals capable of performing ECPR are limited, as they require highly trained personnel, specialized equipment, and well-organized systems that are not available in every healthcare district. Furthermore, the effectiveness of ECPR is highly dependent on minimizing “low-flow time.” Consequently, the chance of receiving ECPR is strongly influenced by the geographic location of arrest, leading to regional disparities and inequities in access to this potentially beneficial therapy [14,15,16,17].

Among the various approaches discussed, two strategies can be proposed and implemented to address these barriers. The first is a two-stage hospital model, in which multiple ECPR-initiation hospitals are distributed within a region; patients undergo cannulation at the nearest initiation site and are then transferred to a tertiary center [18,19]. The second is prehospital ECPR, in which a team is dispatched to the scene—commonly via a physician-staffed ambulance—to initiate cannulation before transport [20,21,22,23]. While both approaches may shorten time to extracorporeal membrane oxygenation (ECMO) initiation, their comparative impact on regional geographic accessibility has rarely been evaluated. Geographic coverage was selected as the primary outcome because ECPR effectiveness is critically time-dependent and the probability of receiving timely ECPR is largely determined by the geographic proximity of patients to ECPR-capable facilities. In this simulation-based study, geographic coverage served as the most direct and practical measure of each strategy’s ability to deliver ECPR within the recommended 60 min window. It should be noted that geographic coverage represents potential accessibility only and does not imply actual ECPR delivery, improved survival, or confirmed clinical benefit. However, direct comparison of these system configurations in real-world settings is limited by ethical and logistical constraints. Therefore, geographic information system (GIS)-based simulation provides a pragmatic framework for evaluating regional accessibility using real EMS transport data without disrupting existing emergency care systems.

The aim of this study was to evaluate, using a GIS–based simulation, which of the two system configurations—the two-stage hospital model or the prehospital ECPR model—would provide broader access to timely ECPR among previously observed witnessed cardiac-origin OHCA cases in Nara Prefecture. The analysis was retrospective, based on actual EMS data, to quantify how each strategy might affect geographic ECPR coverage within the existing regional system.

## 2. Materials and Methods

### 2.1. Study Design

This retrospective, GIS-based simulation study was conducted to evaluate potential accessibility to ECPR among witnessed, cardiac-origin OHCA cases in Nara Prefecture, Japan, between 1 September 2017 and 31 December 2022. Two system configurations (hospital-based and prehospital ECPR deployment) were compared using real-world EMS records. The simulation was focused on geographic feasibility and theoretical accessibility to ECPR under each configuration. Analyses of resource allocation, operational cost, or clinical outcome optimization were beyond the scope of this geographic modeling framework.

Ethical approval was obtained from the Nara Medical University Ethics Committee (approval No. 3863; approved on 8 October 2024), and the requirement for informed consent was waived owing to the retrospective nature of the study.

### 2.2. Study Setting

**Figure 1:**
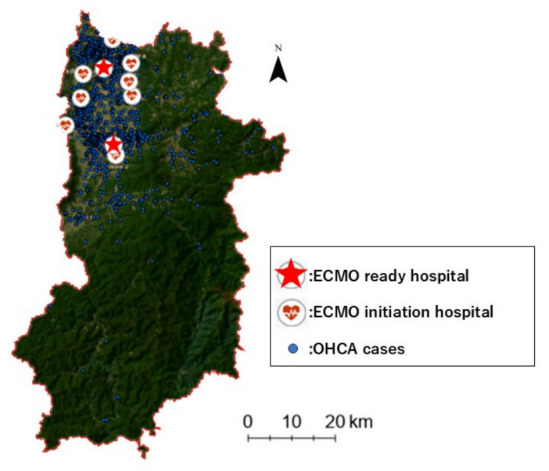
Geographic distribution of OHCA cases and hospital facilities in Nara Prefecture. Blue dots represent OHCA cases. Star icons indicate the two ECMO-ready hospitals currently capable of performing ECPR, which are also part of the chest-pain network. Heart icons indicate the remaining chest-pain network hospitals with 24/7 cardiology coverage designated as potential ECPR-initiation sites. Abbreviations: OHCA: out-of-hospital cardiac arrest; ECMO: extracorporeal membrane oxygenation.

Nara Prefecture, located in western Japan, has a population of approximately 1.3 million, with nearly 90% residing in the northwestern basin (Figure 1). This prefecture encompasses both urban basins and mountainous rural regions, resulting in substantial heterogeneity in EMS transport times.

During the study period, only two hospitals—Nara Prefecture General Medical Center and Nara Medical University Hospital—operated 24/7 as ECMO-ready centers. The latter also runs a physician-staffed ambulance capable of providing advanced on-scene resuscitation, although field ECMO initiation had not yet been implemented.

According to the Nara Prefecture Protocols for Transport and Acceptance of Patients [24], 10 hospitals are designated under the chest-pain network as facilities providing 24-h interventional cardiology coverage for acute coronary syndrome, including the two ECMO-ready hospitals.

### 2.3. Study Population

This study included adults (≥18 years) with witnessed cardiac-origin OHCA occurring in Nara Prefecture between 1 September 2017 and 31 December 2022. Data were obtained from the EMS records of all municipal fire departments in the prefecture.

Patients were excluded if they met any of the following criteria: (1) age ≤ 17 years, (2) inconsistent or invalid data, (3) return of spontaneous circulation (ROSC) before or on EMS arrival, (4) unwitnessed arrest, or (5) non-cardiac etiology.

All eligible patients were included, and no sample size calculation was performed.

### 2.4. Simulation Design and Time Parameters

Two ECMO implementation strategies were modeled for comparison: (1) a two-stage hospital model, in which chest-pain network hospitals with 24/7 cardiology coverage were designated as potential ECPR-initiation hospitals; and (2) a prehospital ECPR model, in which physician-staffed ambulances were dispatched from the two existing ECMO-ready hospitals to the scene to perform cannulation before transport.

Geographic accessibility for each strategy was evaluated using a GIS-based isochrone analysis, which was performed using ArcGIS Pro (version 3.2; ESRI, Redlands, CA, USA), incorporating 2024 OpenStreetMap road-network and speed-limit data. Road data were then supplemented by the 2024 Nara Prefectural Road Registry, applying fixed average travel speeds per road class. Traffic congestion and elevation effects were excluded. Isochrone analysis was employed to determine and visualize areas reachable from a specific starting point within a given travel time.

The time window between patient collapse and ECMO pump-on was set as 60 min based on international consensus recommendations [25,26,27,28]. EMS records provided time and location data, and arrest sites were geocoded for analysis. Service areas were generated using a road network dataset. To ensure that all assumed time components were consistent with realistic operational conditions for both models in the Japanese EMS context, the 60 min time window was divided into subcomponents based on empirical data, institutional practice, and previous literature.

#### 2.4.1. Two-Stage Hospital Model Subcomponents

For hospital-based ECPR initiation, the following time intervals were assumed:

- Call to EMS arrival: 10 min, based on the prefecture’s average call-to-scene interval (10.4 min [28]).
- On-scene EMS activity: 20 min, reflecting the local median of 17 min but rounded conservatively to account for potential delays. This is also based on previous studies showing that prolonged on-scene resuscitation beyond 20 min is associated with decreased survival in OHCA cases without ROSC [29,30].
- Hospital arrival to ECMO pump-on: 15 min, based on institutional experience at Nara Medical University Hospital, where door-to-ECMO initiation is typically achieved within 15 min, consistent with organized ECPR program benchmarks [31].

The remaining allowable time for transport from scene to hospital was therefore 15 min (60–10–20–15 = 15), defining the maximum travel-time threshold for the coverage area in this model.

#### 2.4.2. Prehospital ECPR Model Subcomponents

This model extends the existing ECPR capability of the two ECMO-ready hospitals in Nara Prefecture, which already perform in-hospital ECPR with trained personnel, established protocols, and dedicated ECMO equipment. In this scenario, experienced ECMO teams were assumed to be deployed directly to the field using physician-staffed ambulances, thereby extending hospital-based ECPR capacity into the prehospital setting. The following time intervals were assumed:

- Although EMS typically arrives at the scene within approximately 10 min after the emergency call, the prehospital ECPR team was modeled as being dispatched separately from one of the ECMO-ready base hospitals once the case was identified as a witnessed cardiac arrest.
- Call-to-dispatch preparation: 10 min, representing the time required for assembling the ECMO team and loading equipment prior to departure.
- On-scene preparation for cannulation: 10 min, based on in-hospital preparation times for sterile setup and equipment arrangement under current ECMO protocols at Nara Medical University Hospital.
- Cannulation-to-ECMO pump-on: 15 min, consistent with institutional benchmarks and previously reported intervals in organized ECPR programs [30].

The remaining allowable travel time from the base hospital to the scene was therefore 25 min (60 − 10 − 10 − 15 = 25), defining the travel-time threshold for the prehospital ECPR scenario.

The 10 min team preparation period was assumed to occur concurrently with EMS response to avoid double-counting within the 60 min total.

This prehospital model assumed immediate recognition of included witnessed cardiac-origin OHCA cases and did not incorporate dispatch latency due to triage or communication delays, as the analysis was focused on geographic feasibility rather than operational efficiency.

### 2.5. Patient Demographics and EMS Operational Characteristics

Patient demographics and EMS operational characteristics, such as age, sex, bystander CPR, initial rhythm, airway management, intravenous access, epinephrine administration, and EMS time intervals (call-to-scene, on-scene, and transport times), were summarized to describe the study cohort.

### 2.6. Outcome

The outcome was coverage rate, defined as the proportion of witnessed, cardiac-origin OHCA cases located within the estimated service area that allowed ECPR initiation within 60 min from cardiac arrest to ECMO pump-on.

For each strategy, geographic coverage was determined using GIS-based isochrone analysis, which was used to estimate travel times between the arrest site and the nearest ECPR-initiation hospital or base hospital operating-physician-staffed ambulances.

### 2.7. Statistical Analysis

Geographic coverage rates were calculated as the proportion of eligible OHCA cases within the simulated service areas for each strategy.

Comparisons between the two strategies (two-stage hospital model vs. prehospital ECPR model) were performed using McNemar’s test for paired proportions, with 95% confidence intervals (CIs). Patient and EMS characteristics were summarized as medians (interquartile ranges [IQR]) or counts (%).

A prespecified subgroup analysis was performed to assess geographic coverage among cases presenting with a shockable initial rhythm (ventricular fibrillation or pulseless ventricular tachycardia), who represent the most clinically plausible candidates for ECPR. Coverage rates in this subgroup were calculated using the same methods as the primary analysis.

All analyses were performed using EZR (version 1.55; Saitama Medical Center, Jichi Medical University, Japan), a graphical user interface for R (version 4.2.2) [32].

### 2.8. Sensitivity Analysis

To assess the robustness of the prehospital ECPR model, a sensitivity analysis was performed by varying the combined on-scene preparation and cannulation time by 5%, 10%, and 15% above the base case (25 min), thus corresponding to 26.25 min, 27.5 min, and 28.75 min, respectively. For the two-stage hospital model, the door-to-ECMO time was varied from −15% to +15% relative to the base case (15 min) in increments of 5%, corresponding to 12.75, 13.5, 14.25, 15.75, 16.5, and 17.25 min, to identify the conditions under which this model could match or exceed the geographic coverage of the prehospital ECPR model.

## 3. Results

### 3.1. Case Selection and Study Cohort

During the study period (September 2017–December 2022), a total of 5897 OHCA cases occurred in Nara Prefecture. After excluding pediatric patients aged ≤17 years (n = 62) and cases with incomplete or inconsistent data (n = 356), 5479 cases remained. Among these, 4003 cases were excluded for the following reasons: prehospital ROSC confirmed before or upon EMS arrival (n = 333), unwitnessed cardiac arrest (n = 3135), and cardiac arrest of non-cardiac etiology (n = 535).

**Figure 2:**
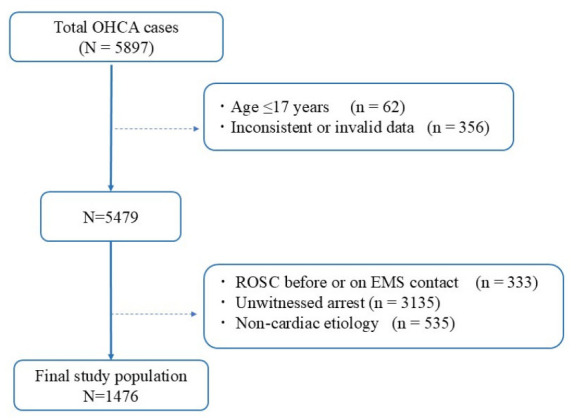
Flow diagram of study cohort selection. Abbreviations: EMS: Emergency Medical Services; OHCA: out-of-hospital cardiac arrest; ROSC: return of spontaneous circulation.

Consequently, a total of 1476 cases of witnessed presumed cardiac-origin OHCA were included in the final analysis (Figure 2). These cases represented the cohort eligible for ECPR within the study region.

### 3.2. Baseline Characteristics of Included Cases

Baseline characteristics of the study population (n = 1476) are shown in Table 1. The median age was 82 years (IQR, 72–88), and more than half (59.9%, n = 881) were male. Bystander chest compressions were performed in 807 patients (54.7%). On EMS arrival, 229 patients (15.5%) presented with a shockable initial rhythm, while the majority exhibited non-shockable rhythms (PEA or asystole). Regarding EMS interventions, advanced airway management was performed in 1161 patients (78.7%), intravenous access was established in 663 (46.3%), and epinephrine was administered in 636 (43.1%). Time intervals for EMS activity were as follows: call-to-scene arrival, median 9.0 min (IQR, 7.0–11.0); on-scene time, median 17.0 min (IQR, 13.0–22.0); and transport time from scene to hospital, median 10.0 min (IQR, 6.0–14.0). Overall, ROSC was achieved in 243 patients (16.5%), and a favorable neurological outcome (CPC 1–2) was observed in 73 patients (4.4%).

### 3.3. Baseline Geographic Coverage Under the Current System

**Figure 3:**
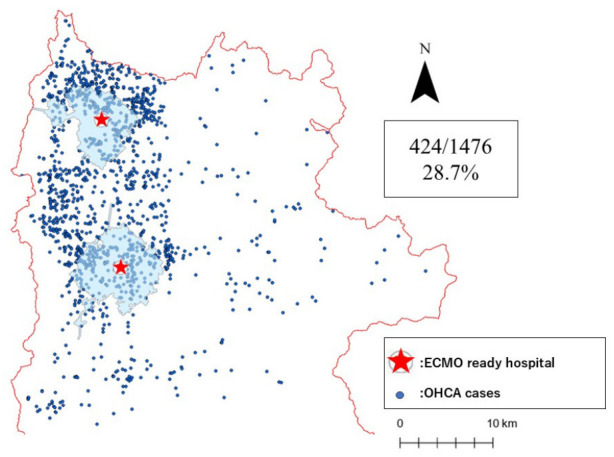
Geographic coverage of OHCA cases by the two existing ECMO-ready hospitals in Nara Prefecture. The blue-shaded areas represent regions reachable within 60 min from the two ECMO-ready hospitals (indicated by star icons), covering 424 of 1476 witnessed cardiac-origin OHCA cases (28.7%). Abbreviations: ECMO, extracorporeal membrane oxygenation; OHCA, out-of-hospital cardiac arrest.

Under the current system with only two ECMO-ready hospitals, geographic coverage was limited to 28.7% (n = 424) (Figure 3).

### 3.4. Comparison of Hospital-Based Versus Prehospital ECPR Models

**Figure 4:**
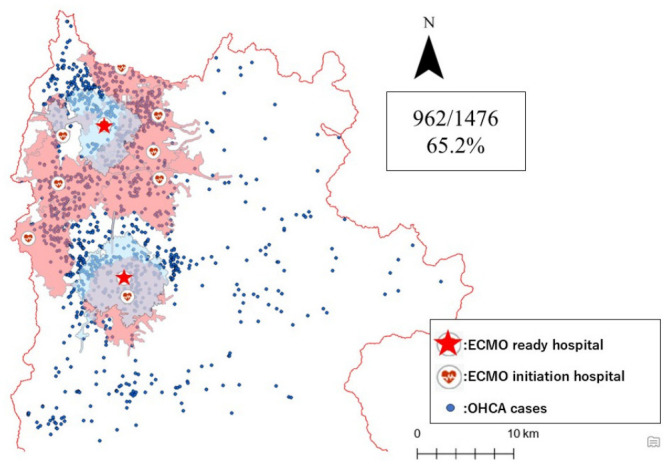
ECPR accessibility with the two-stage hospital model (ECPR-initiation hospitals). The light-red shaded areas indicate the combined catchment zones of all ECPR-initiation hospitals under the two-stage model, while the blue-shaded areas show the 60 min coverage by the two existing ECMO-ready hospitals (indicated by star icons) for reference. In total, 962 of 1476 OHCA cases (65.2%) were located within 60 min accessibility under this model. Abbreviations: ECPR, extracorporeal cardiopulmonary resuscitation; ECMO, extracorporeal membrane oxygenation; OHCA, out-of-hospital cardiac arrest.

**Figure 5:**
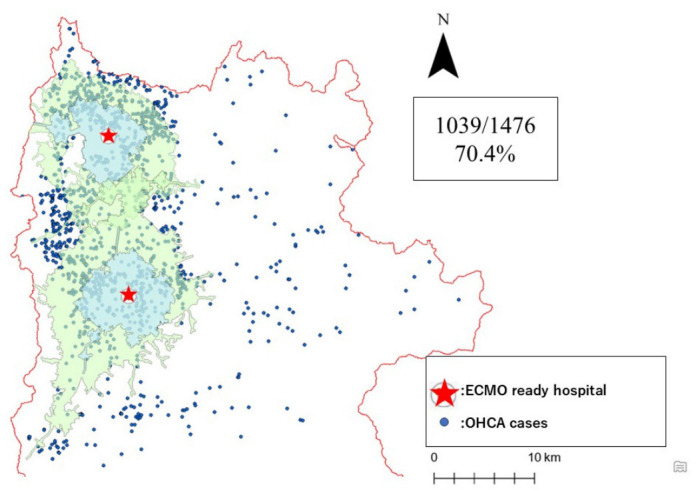
Geographic coverage of OHCA cases under the physician-staffed-ambulance-based prehospital ECPR strategy. The light-green shaded areas represent regions reachable within the 60 min therapeutic window under the physician-staffed ambulance strategy, while the blue-shaded areas indicate the 60 min coverage by the two existing ECMO-ready hospitals (indicated by star icons) for comparison. This model covered 1039 of 1476 included OHCA cases (70.4%). Abbreviations: ECPR, extracorporeal cardiopulmonary resuscitation; ECMO, extracorporeal membrane oxygenation; OHCA, out-of-hospital cardiac arrest.

When the two-stage hospital model was applied—designating additional hospitals as ECPR-initiation sites—coverage increased substantially to 65.2% (n = 962, Figure 4). Further improvement was achieved in the prehospital ECPR model, with coverage expanding to 70.4% of cases (n = 1039, Figure 5). The difference in coverage between the two models was statistically significant (*p* < 0.001, McNemar’s test), with an absolute increase of 5.2% (95% CI, 2.3–8.2%). Paired contingency tables illustrating the coverage relationships between strategies are presented in Table 2. All 424 cases covered under the current system were also covered under the two-stage hospital model, while an additional 538 cases were newly covered (Table 2A). When comparing the two-stage hospital model with the prehospital ECPR model, 289 additional cases were covered by the prehospital model that were not covered by the two-stage model, and 212 cases covered by the two-stage model were not covered by the prehospital model (Table 2B).

Among the 156 included cases with a shockable initial rhythm (10.6% of the total cohort), geographic coverage was as follows: current system, 30.1% (47/156); two-stage hospital model, 68.6% (107/156); prehospital ECPR model, 75.0% (117/156). These findings were directionally consistent with those of the primary analysis, suggesting that the geographic patterns observed are not materially altered when the analysis is restricted to the most clinically plausible ECPR candidates.

### 3.5. Residual Uncovered Areas

Despite the improved coverage under both alternative models, 514 cases (34.8%) remained uncovered under the two-stage hospital model and 437 cases (29.6%) under the prehospital ECPR model. Furthermore, 225 cases (15.2% of the total cohort) remained outside the coverage area of both models. These uncovered cases were concentrated in the eastern and southern mountainous regions of Nara Prefecture (Figure 4 and Figure 5), reflecting the geographic limitations inherent to both hospital-based and prehospital strategies.

### 3.6. Results of Sensitivity Analysis

**Figure 6:**
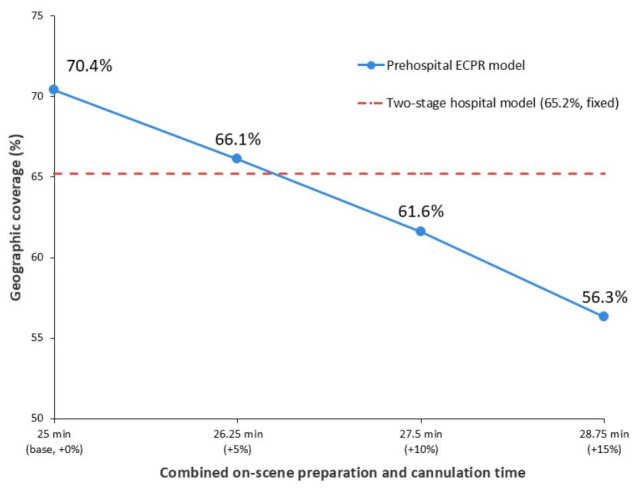
Sensitivity analysis of geographic coverage rates for the prehospital ECPR model according to variations in combined on-scene preparation and cannulation time. Data points represent discrete simulation results. The dashed red line indicates the fixed coverage rate of the two-stage hospital model (65.2%).

**Figure 7:**
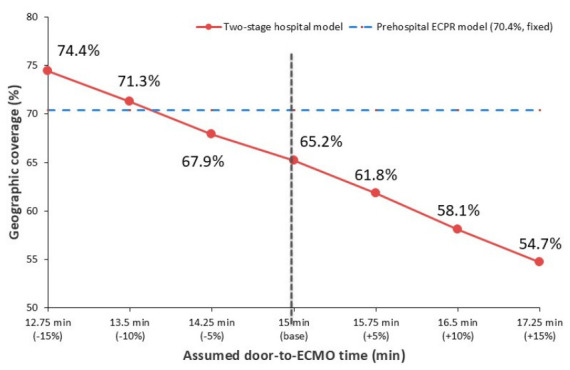
Sensitivity analysis of geographic coverage rates for the two-stage hospital model according to variations in door-to-ECMO time (base case: 15 min). The dashed blue line indicates the fixed base-case coverage of the prehospital ECPR model (70.4%). The vertical dotted line indicates the base case (15 min). Data points represent discrete simulation results.

Sensitivity analysis demonstrated that the prehospital ECPR model maintained its geographic advantage over the two-stage hospital model when the combined on-scene preparation and cannulation time remained within 5% above the base case. However, when this time increased by ≥10%, the two-stage hospital model provided superior coverage (Figure 6). For the two-stage hospital model, sensitivity analysis showed that this model exceeded the base-case coverage of the prehospital ECPR model only when the door-to-ECMO time was reduced by ≥10% below the base case (i.e., to ≤13.5 min) (Figure 7).

## 4. Discussion

This study showed that a mobility-focused prehospital ECPR model extended potential access beyond what was achievable through hospital expansion, particularly in resource-limited areas. The interpretation and implications of these findings are discussed in this section.

Two ECPR implementation scenarios in witnessed cardiac-origin OHCA cases in Nara Prefecture were examined: a “two-stage hospital model,” involving designation of multiple ECPR-initiation hospitals, and a “prehospital ECPR model,” involving use of physician-staffed ambulances. Simulation analysis demonstrated that, under the current system with only two ECMO-ready hospitals, the proportion of cases eligible for ECPR was limited to 28.7%. Expanding ECPR initiation to multiple hospitals markedly increased coverage to 65.2%, while further improvement to 70.4% was achieved when both ECMO-ready hospitals were assumed to provide prehospital ECPR via physician-staffed ambulances. The difference between the two strategies was statistically significant (*p* < 0.001, McNemar’s test), indicating that the prehospital approach provided access to cases that would not be covered even if all candidate hospitals were designated as ECPR-initiation sites. Furthermore, the prehospital model enables EMS providers to continue high-quality resuscitation on scene while a physician-led team stands ready to initiate ECMO if ROSC is not achieved. This dual pathway preserves both opportunities—conventional resuscitation success and timely ECPR initiation—and may mitigate CPR quality deterioration or delays associated with transport, thereby improving survival, as suggested by recent studies [19,33]. Since establishing additional ECPR-initiation hospitals requires considerable financial and human resources [34,35,36], we avoided making economic claims and instead noted that optimizing prehospital strategies may represent a more practical approach to expanding access, particularly in regions where resource and geographic constraints limit the feasibility of increasing hospital-based ECPR capacity. Nevertheless, the three strategies differ substantially in their implementation burden beyond geographic coverage alone; a formal cost-effectiveness comparison remains an important area for future research.

### 4.1. Interpretation of Findings

While prehospital ECPR is theoretically highly efficient and flexible, multiple challenges remain in its implementation. First, advanced procedures such as cannulation and ECMO circuit establishment must be performed safely and reliably in the field, which requires specialized training and thorough preparation of the response team [21,37]. In the simulation by Song et al. [37], prehospital cannulation time was set at 22 min as the base case, with 27 min representing a pessimistic scenario. Our base case assumption of 25 min already approaches this pessimistic threshold; yet, the prehospital ECPR model maintained its geographic advantage up to approximately 26.25 min. However, when preparation and cannulation times increased by ≥10%, the two-stage hospital model provided superior coverage, highlighting the critical importance of team training and standardized protocols for reliable prehospital ECPR implementation. Second, rapid identification and dispatch of included cases demand improved triage accuracy and standardized on-scene decision-making. Third, physician-staffed ambulance systems entail substantial investment in personnel, equipment, and operational infrastructure, and must be carefully coordinated with existing EMS systems. The required team typically includes at minimum two consultant-level specialists and a clinical perfusionist [22] and must be capable of performing cannulation and ECMO circuit establishment safely under conditions far less controlled than a hospital setting.

Sensitivity analysis confirmed that the geographic advantage of the prehospital ECPR model is robust to plausible variation in the time parameters of the two-stage hospital model. The two-stage hospital model exceeded the base-case coverage of the prehospital ECPR model only when the door-to-ECMO time was reduced by ≥10% below the base case (i.e., to ≤13.5 min), a level of performance that would be challenging to sustain consistently across all designated chest-pain network hospitals.

Despite these challenges, a physician-staffed ambulance strategy may still represent a practical means of expanding ECPR accessibility by flexibly extending coverage without duplicating hospital infrastructure.

### 4.2. Geographic Disparities and Equity

An important finding of this study was that certain eastern and southern mountainous regions of Nara Prefecture remained outside the ECPR coverage area, even under the optimized two-stage hospital model and prehospital strategy. These areas lack nearby large hospitals suitable for ECPR initiation, and prolonged transport times make ECPR initiation within 60 min unlikely. Because these mountainous regions also include small but inhabited communities (Figure 4 and Figure 5), such limited access represents a geographic disadvantage in life-saving opportunities. However, regional disparities in access to advanced interventions such as ECPR are not unique to Nara and have been described in other countries, where some degree of limited coverage is considered unavoidable owing to geographic constraints [15].

### 4.3. Implementation Challenges

Nevertheless, recognizing these underserved areas is valuable, as it highlights the need for different strategies to mitigate such disparities. Geographic simulation analyses, as performed in this study, provide important insights for identifying gaps and informing region-specific system design. Although helicopter-based ECPR could theoretically expand access in mountainous regions, this approach was not modeled in the present simulation and remains an area for future investigation [38]. Moreover, implementation would require larger aircraft and specialized logistics [39,40]. In the specific context of the Nara Prefecture, a hybrid strategy may be worth consideration: given the limited cabin space of Japanese physician-staffed helicopters and the impracticality of carrying ECMO equipment on every flight, the helicopter team could initiate cannulation at the scene while a ground-based physician-staffed ambulance carrying ECMO equipment arrives simultaneously. This approach adapts helicopter-based ECPR concepts reported elsewhere [38] to the operational constraints of the Japanese prehospital setting and warrants further investigation.

### 4.4. Policy Implications

Furthermore, the simulation-based framework presented in this study shows how universally available EMS operational data can be integrated with GIS modeling to identify region-specific and operationally practical solutions. Because this approach relies on generalizable data and methods, it can be readily adapted to other prefectures or countries with differing resources and geography. By quantifying time-dependent accessibility, each region can objectively evaluate which ECPR strategy—hospital expansion, prehospital deployment, or hybrid models—would be most effective under its own conditions. In the present study, as illustrated in Table 2B, 289 cases were covered exclusively by the prehospital model and 212 cases exclusively by the two-stage hospital model, suggesting that a hybrid approach combining both strategies may offer the broadest potential geographic accessibility for ECPR in Nara Prefecture. This flexibility underscores the framework’s value as a practical decision-support tool for policymakers aiming to improve access to advanced resuscitation care.

### 4.5. Limitations

First, factors such as traffic conditions along the transport route, geographic characteristics, and the experience and capability of EMS crews may substantially influence the actual time to hospital arrival. Because traffic congestion and elevation effects were explicitly excluded from the GIS-based isochrone model, the simulated travel times should be interpreted as best-case estimates under idealized road conditions. Caution is therefore warranted when extrapolating these findings to other regions or countries with different road networks, traffic patterns, or geographic characteristics.

Second, this simulation assumed that the nearest ECPR-capable hospital or base hospital is always available to respond; however, in real-world settings, the nearest facility may not always be accessible owing to competing emergency cases, bed occupancy, or operational constraints, which may result in an overestimation of geographic coverage. Furthermore, this study did not consider the simultaneous occurrence of multiple OHCA events. In practice, the number of available ambulances and hospital beds required to achieve the reported coverage rates was not assessed, and resource competition may further limit real-world applicability.

Third, the assumptions regarding future ECPR operations are uncertain. The time intervals applied in the two-stage hospital model and prehospital ECPR model (e.g., on-scene time, cannulation time) were based on limited prior studies and guidelines, and these assumptions may vary in actual implementation. Furthermore, the two-stage hospital model assumed that all chest-pain network hospitals could function as ECPR-initiation sites within the same door-to-ECMO time threshold of 15 min. In practice, the ability to perform rapid cannulation within this interval would depend on institutional readiness, including the availability of trained personnel, dedicated ECMO equipment, and established activation protocols, which may vary considerably across designated hospitals.

Fourth, this study was focused on the “geographic accessibility” of ECPR, without considering patients’ medical eligibility (e.g., age, comorbidities, or prognostic factors). Indeed, the median age of the study cohort was 82 years, and many cases would likely fall outside the clinical indications for ECPR. Therefore, the coverage rates estimated in this study should be interpreted as idealized values and may differ from actual implementation rates. Furthermore, geographic coverage does not encompass service readiness; actual ECPR delivery depends on clinical complexity, equipment availability, staff expertise, and institutional preparedness, all of which may vary substantially across hospitals and settings. However, in the two-stage hospital model, ECPR-initiation hospitals were assumed to perform cannulation only, with subsequent transfer to ECMO-ready hospitals for definitive management, which partially mitigates concerns regarding variability in institutional preparedness.

Fifth, some cases may achieve ROSC through advanced procedures by EMS personnel, such as epinephrine administration or airway management, but this study did not account for these effects. Consequently, patients who did not actually require ECPR may have been included.

Finally, simulated travel times derived from the GIS-based isochrone model were not validated against actual EMS transport intervals in Nara Prefecture, and the prehospital model assumed immediate case identification and uniform dispatch preparation time; these simplifications could overestimate achievable geographic coverage, particularly in rural areas or those with heavy traffic congestion. Although a formal validation of GIS-derived travel times against actual EMS transport intervals was not performed in the present study, the empirically observed median scene-to-hospital transport time of 10.0 min (IQR 6.0–14.0) in our cohort is broadly consistent with the modeled travel time thresholds applied in the GIS analysis, supporting the plausibility of our coverage estimates. Although the road network data were derived from a 2024 map dataset, no major changes in arterial roads or expressway infrastructure occurred during the study period, and thus the data are unlikely to have substantially affected the study results.

Despite these limitations, this study provides practical and policy-relevant insights into ECPR system development by applying geographic simulations based on real OHCA case data. Future work should incorporate validation using observed EMS time intervals and account for operational delays in case identification and dispatch when modeling prehospital ECPR.

## 5. Conclusions

This GIS-based simulation study demonstrated that a physician-staffed ambulance strategy for prehospital ECPR could achieve broader geographic coverage compared with expanding hospital-based ECPR facilities, without requiring additional fixed hospital infrastructure. However, sensitivity analysis revealed that this geographic advantage was contingent on achieving on-scene procedures within the assumed time parameters. These findings may inform region-specific planning for ECPR system design.

## Figures and Tables

**Table 1:** Patient characteristics.

Characteristic	Value (All Patients n = 1476)
Patient age, years	82.0 (72.0–88.0)
Male, n (%)	881 (59.9)
Bystander chest compressions, n (%)	807 (54.7)
Shockable rhythm at EMS arrival, n (%)	229 (15.5)
Advanced airway management by EMS, n (%)	1161 (78.7)
Intravenous access by EMS, n (%)	663 (46.3)
Epinephrine administration by EMS, n (%)	636 (43.1)
Time from emergency call to EMS arrival at scene, min	9.0 (7.0–11.0)
EMS on-scene time, min	17.0 (13.0–22.0)
Scene-to-hospital transport time, min	10.0 (6.0–14.0)
ROSC, n (%)	243 (16.5)
CPC 1–2, n (%)	73 (4.4)

Values are reported either as n (%) or median (interquartile range, IQR). Abbreviations: ROSC, return of spontaneous circulation; CPC, cerebral performance category.

**Table 2:** Paired contingency tables of geographic coverage between ECPR strategies.

**(A) Current System Versus Two-Stage Hospital Model**			
	**Two-Stage: Covered**	**Two-Stage: Not Covered**	**Total**
Current: Covered	424 (28.7%)	0 (0.0%)	424 (28.7%)
Current: Not covered	538 (36.4%)	514 (34.8%)	1052 (71.3%)
Total	962 (65.2%)	514 (34.8%)	1476 (100%)
**(B) Two-Stage Hospital Model Versus Prehospital ECPR Model**			
	**Prehospital: Covered**	**Prehospital: Not Covered**	**Total**
Two-stage: Covered	750 (50.8%)	212 (14.4%)	962 (65.2%)
Two-stage: Not covered	289 (19.6%)	225 (15.2%)	514 (34.8%)
Total	1039 (70.4%)	437 (29.6%)	1476 (100%)

*p* < 0.001 (McNemar’s test). Values are presented as n (%). Percentages are calculated based on total included cases (n = 1476). Abbreviations: ECPR, extracorporeal cardiopulmonary resuscitation.

## Data Availability

The data that support the findings of this study are available from the Nara Wide Area Fire Department, Nara City Fire Department and Ikoma City Fire Department, but restrictions apply to the availability of these data, which were used under license for the current study and are not publicly available. Data are, however, available from the authors upon reasonable request and with permission of the Nara Wide Area Fire Department, Nara City Fire Department and Ikoma City Fire Department.

## Abbreviations

AED	Automated External Defibrillator
CPC	Cerebral Performance Category
CPR	Cardiopulmonary Resuscitation
ECPR	Extracorporeal Cardiopulmonary Resuscitation
ECMO	Extracorporeal Membrane Oxygenation
EMS	Emergency Medical Services
GIS	Geographic Information System
IQR	Interquartile Range
OHCA	Out-of-Hospital Cardiac Arrest
ROSC	Return of Spontaneous Circulation
